# Supplementary material for: Orbital-symmetry effects on magnetic exchange in open-shell nanographenes
Source: Nat Commun. 2023 Aug 9;14:4802. doi: 10.1038/s41467-023-40542-0 (PMC10412602; doi:10.1038/s41467-023-40542-0)
Supplement: Supplementary file 1 — Supplementary Information [file 41467_2023_40542_MOESM1_ESM.pdf]

Supporting Information

Orbital-Symmetry Effects on Magnetic Exchange in Open-Shell Nanographenes

Qingyang Du[1,†], Xuelei Su[1,†], Yufeng Liu[2,†], Yashi Jiang[2,†], Can Li[2], KaKing Yan[1], Recardo Ortiz[3,*], Thomas Frederiksen[3,4,*], Shiyong Wang[2,5,*] and Ping Yu[1,*]

†These authors contributed equally to this work

Contents

1 Solution synthesis **3**

1.1 Synthetic procedures 3

1.2 NMR and mass spectroscopy 7

2 Extended electronic characterization data **13**

2.1 dI/dV and d^2I/dV^2 spectra of **D1** and **D2** 13

2.2 Large range dI/dV spectra and dI/dV mappings of **D1**,**D2 and T** 14

3 Extended theoretical calculations **16**

3.1 Magnetic ground state and first excited spin state of **D1** and **D2** using MFH method 16

3.2 Effect of next-nearest hopping term t_2 16

3.3 Wave function distribution calculated with U = 0 eV and U = 3.5 eV 17

4 Sample preparation and AFM, STM, STS measurements **19**

5 Supplementary References **19**

1 Solution synthesis

Compound **1a** was prepared according to previous approach, giving the same NMR and mass data,[1] while the other starting chemical materials were purchased from TCI, Adamas, SCRC, Bide, and other chemical providers. All starting materials were received without further purification. The solution reactions under air- and moisture-free conditions were conducted by using a sealed Schlenk system under nitrogen atmosphere because some substances are air- or moisture-sensitive. The reaction progress was monitored by thin layer chromatography (TLC) which contains silica-coated glass plates and fluorescence marker F_{254}. Crude reaction products were purified by preparative silica gel chromatography (particle size: 45-75 μm, Greagent). For structure characterization., proton and carbon nuclear magnetic resonance spectra (^{1}H and ^{13}C-NMR, respectively) were carried out at 298 K on a BRUKER NMR instrument, operating at 400 MHz for ^{1}H-NMR and 101 MHz for ^{13}C-NMR. The NMR measurements were carried out in the liquid-state using deuterated chloroform ($CDCl_3$, 99% D, $\delta_{\text{H-NMR}}$ = 7.26 ppm/ $\delta_{\text{C-NMR}}$ = 77.3 ppm). The peak pattern in ^{1}H-NMR spectra is described by commonly used abbreviations: s = singlet, d = doublet, t = triplet and m = multiplet. High-resolution atmospheric pressure chemical ionization MS (HR-APCI-MS) was recorded with the Agilent system.

1.1 Synthetic procedures

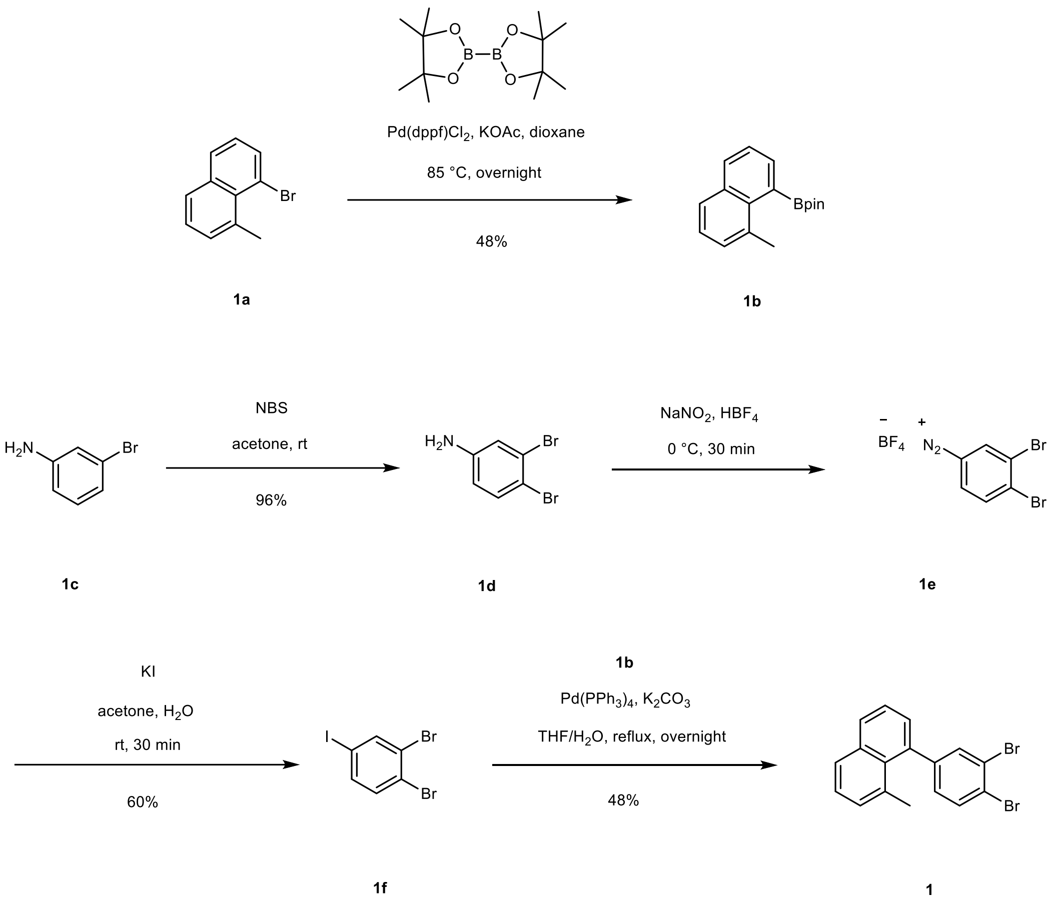

Supplementary Figure 1. Summary of synthetic procedures toward formation of **1**.

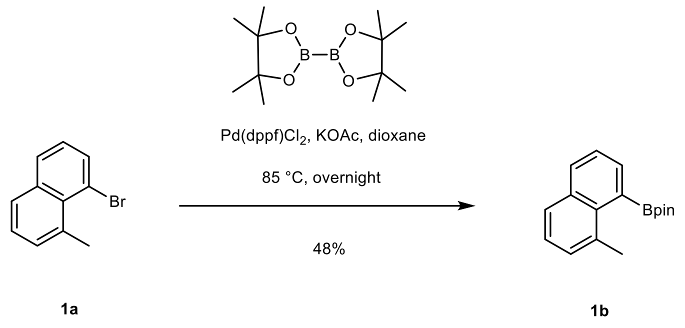

Supplementary Figure 2. Synthesis of **1b**.

Synthesis of **1b**: Compound **1a** (6g, 27.14 mmol, 1 eq), B_2Pin_2 (8.27 g, 32.56 mmol, 1.2 eq), Pd(dppf)Cl_2 (0.397 g, 0.54 mmol, 2 mol %), KOAc (5.327 g, 54.28 mmol, 2 eq) were put in a Schelenk reaction tube and filled with pure N_2 to keep an inert

atmosphere. Then dioxane was added and the reaction was kept at 85°C overnight before cooling to room temperature. The reaction was washed with brine and the organic phase was extracted with DCM. The combined organic phase was dried with Na_2SO_4 and the solvent was evaporated. The mixture was purified through silica gel column (PE : DCM = 2 : 1). Resulted product **1b** (3.49 g, yield: 48 %) was a brown solid.

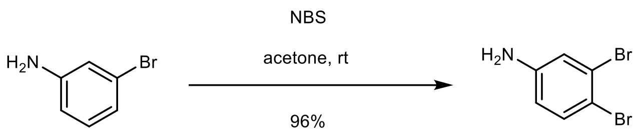

1c **1d**

Supplementary Figure 3. Synthesis of **1d**.

Synthesis of **1d**: NBS (8.18 g, 45.93 mmol, 1eq) was dissolved in acetone and added in portion to the solution of compound **1c** (7.9 g, 45.93 mmol, 1eq) in acetone. The reaction was monitored with TLC. After the end of the reaction, the mixture was purified through silica gel column (pure DCM) and gave **1d** (11.06 g, yield: 96 %) as a light orange solid.

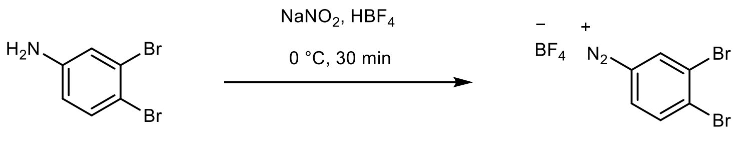

1d **1e**

Supplementary Figure 4. Synthesis of **1e**.

Synthesis of **1e**: Compound **1d** (12 g, 47.83 mmol, 1 eq) was added to HBF_4 (16 ml, 48% wt, 119.58 mmol, 2.5 eq) in 480 ml H_2O and kept under 0°C. $NaNO_2$ (4 g, 57.4 mmol, 1.2 eq) was dissolved in 16 ml H_2O and added dropwise to the above system. The mixture was kept under 0°C for 30 minutes and then the generated precipitation was filtered and washed with 5% HBF_4 aqueous solution, -20°C MeOH and Et_2O respectively. The obtained yellow powder was used directly into next step.

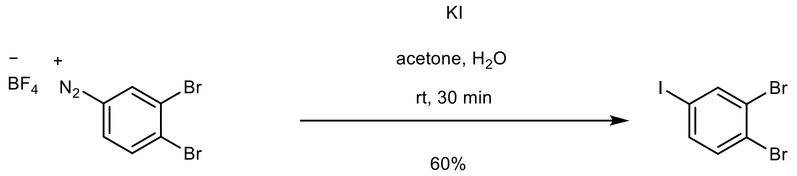

1e **1f**

Supplementary Figure 5. Synthesis of **1f**.

Synthesis of **1f**: KI (7.94 g, 47.83 mmol, 2 eq) was dissolved in acetone and H_2O, and compound **1e** (8.36 g, 23.92mmol, 1 eq) was added in portion. The mixture was stirred at room temperature for 30 minutes. Once the reaction was ended, acetone was removed through evaporation and the left mixture was washed with $Na_2S_2O_3$ aqueous solution and then extracted with DCM. The organic phase was dried with Na_2SO_4 and the

solvent was evaporated. The crude product was purified with column chromatography using PE as eluent to get **1f** with a little 1,2-dibromobenzene in it as a white solid. The obtained product was directly used into the next step.

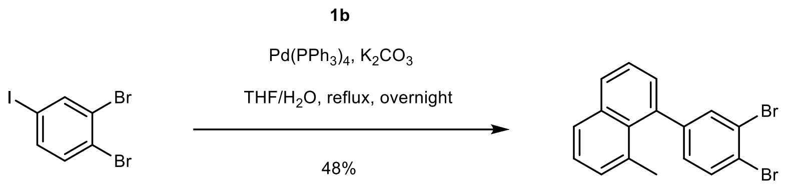

1f 1

Supplementary Figure 6. Synthesis of **1**.

Synthesis of **1**: Compound **1f** (2.78 g, 7.68 mmol, 1eq), **1b** (2.75 g, 10.26 mmol, 1.3 eq), $Pd(PPh_3)_4$ (0.23 g, 0.20 mmol, 2.6 mol%), K_2CO_3 (2.77 g, 20 mmol, 2.6 eq) were put in a Schelenk reaction tube and filled with pure N_2 to keep an inert atmosphere. Then THF/H_2O was added and the reaction was refluxed overnight before cooling to room temperature. The mixture was washed with brine and the organic phase was extracted with DCM. The combined organic phase was dried with Na_2SO_4 and the solvent was evaporated. The mixture was purified through silica gel column (pure PE). Resulted product **1b** (1.38 g, yield: 48%) was a white solid.

1.2 NMR and mass spectroscopy

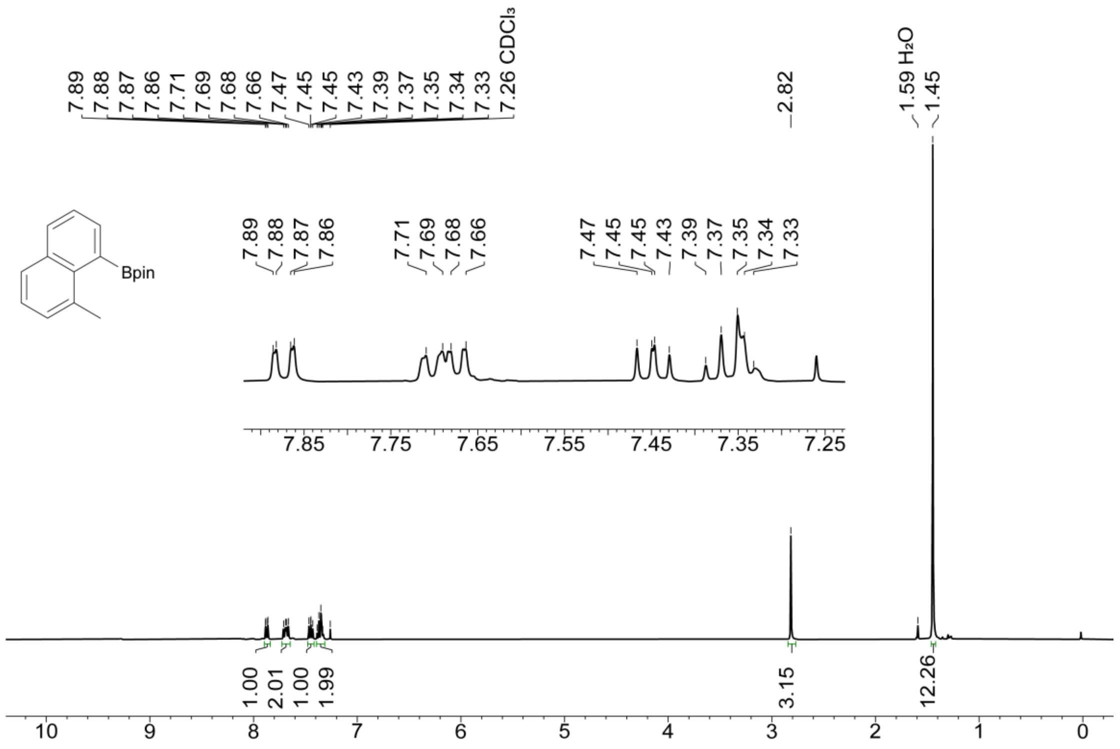

Supplementary Figure 7. Liquid-state ^{1}H NMR spectrum of compound **1b** measured in $CDCl_3$ at room temperature. Frequency: 400 MHz.

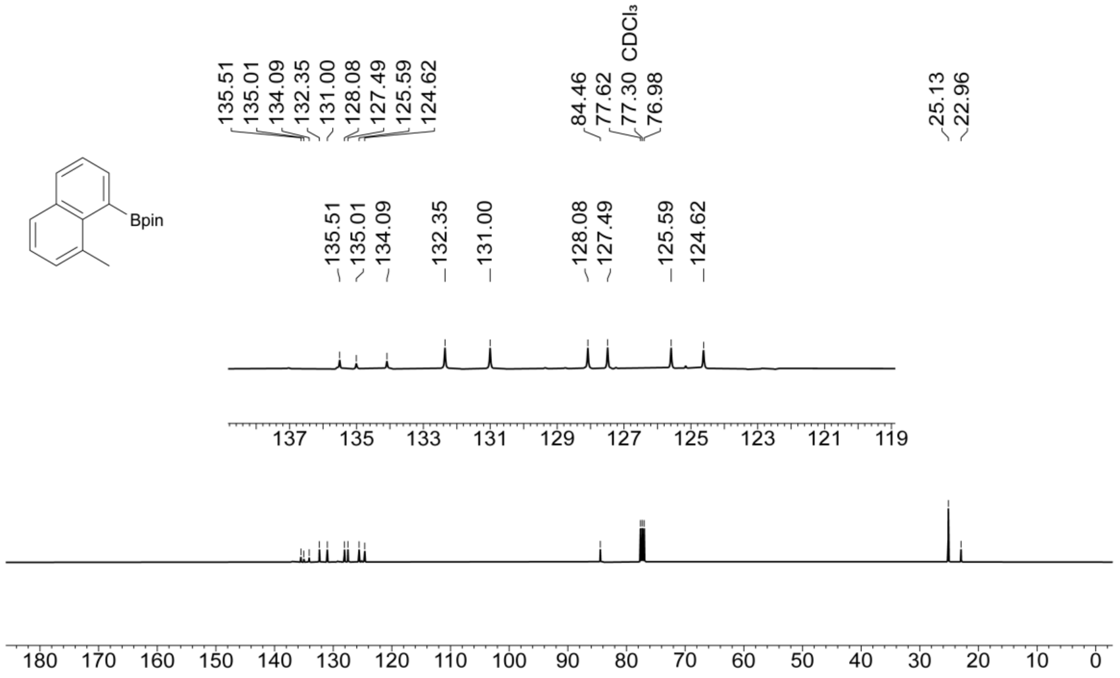

Supplementary Figure 8. Liquid-state ^{13}C NMR spectrum of compound **1b** measured in $CDCl_3$ at room temperature. Frequency: 101 MHz.

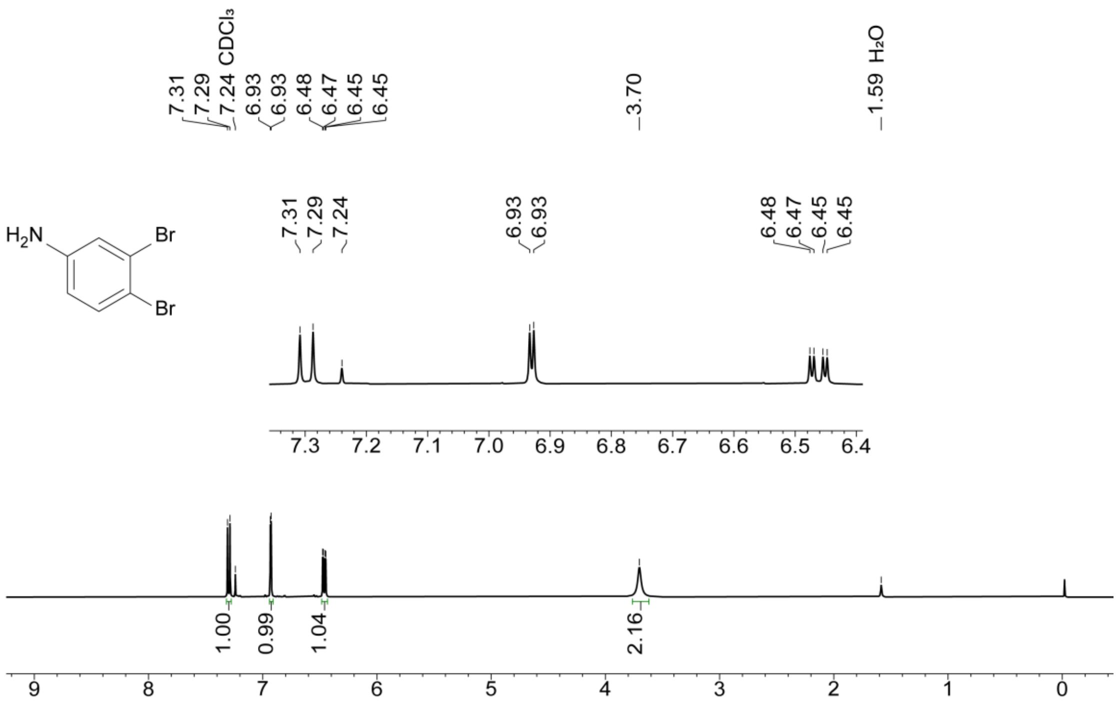

Supplementary Figure 9. Liquid-state ^{1}H NMR spectrum of compound **1d** measured in $CDCl_3$ at room temperature. Frequency: 400 MHz.

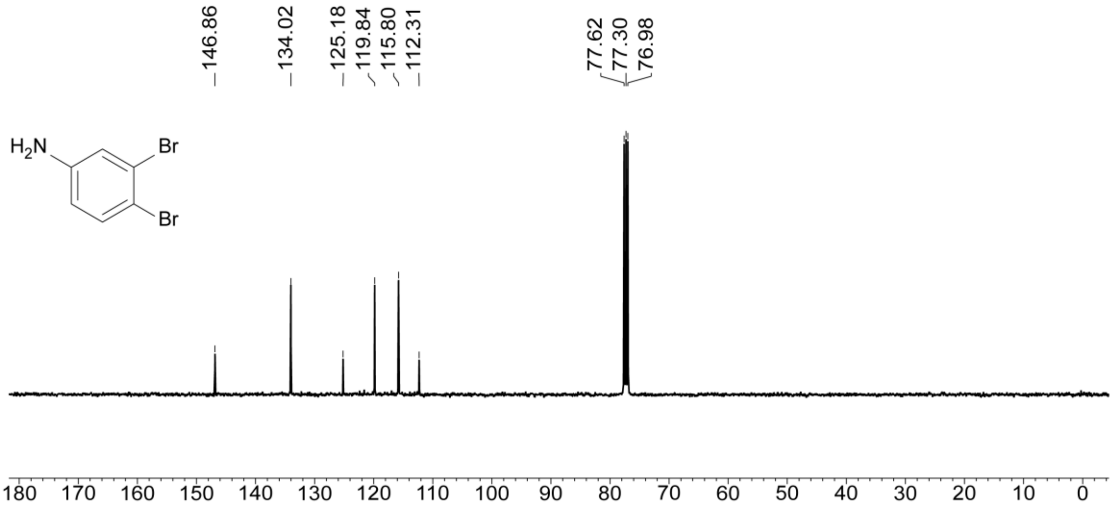

Supplementary Figure 10. Liquid-state ^{13}C NMR spectrum of compound **1d** measured in $CDCl_3$ at room temperature. Frequency: 101 MHz.

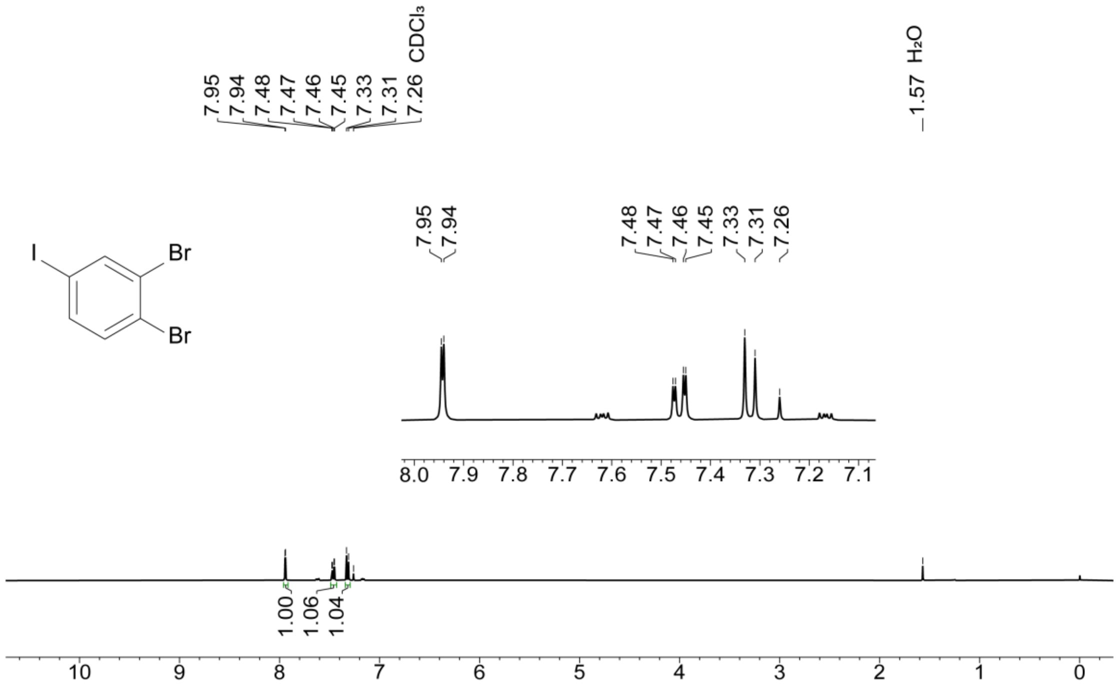

Supplementary Figure 11. Liquid-state ^{1}H NMR spectrum of compound **1f** measured in $CDCl_3$ at room temperature. Frequency: 400 MHz.

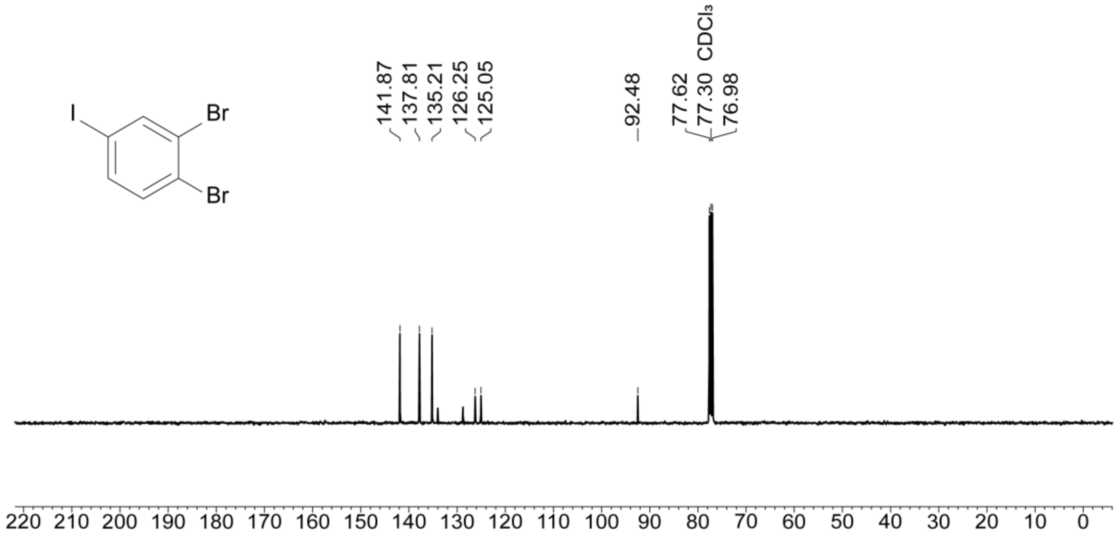

Supplementary Figure 12. Liquid-state ^{13}C NMR spectrum of compound **1f** measured in $CDCl_3$ at room temperature. Frequency: 101 MHz.

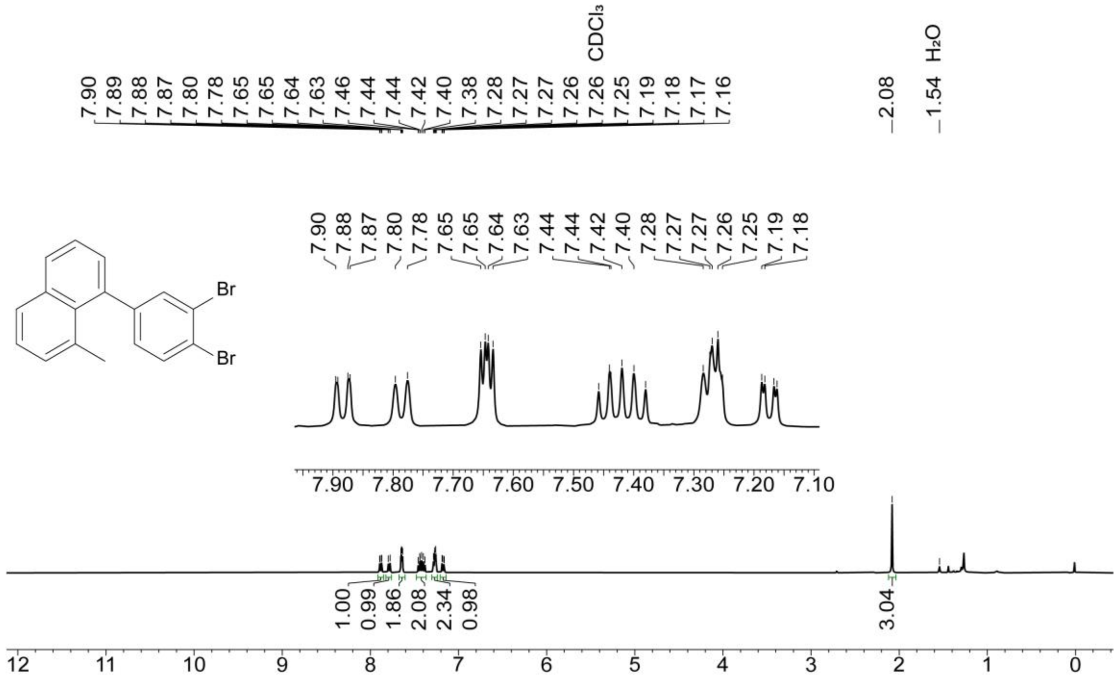

Supplementary Figure 13. Liquid-state ^{1}H NMR spectrum of compound **1** measured in $CDCl_3$ at room temperature. Frequency: 400 MHz.

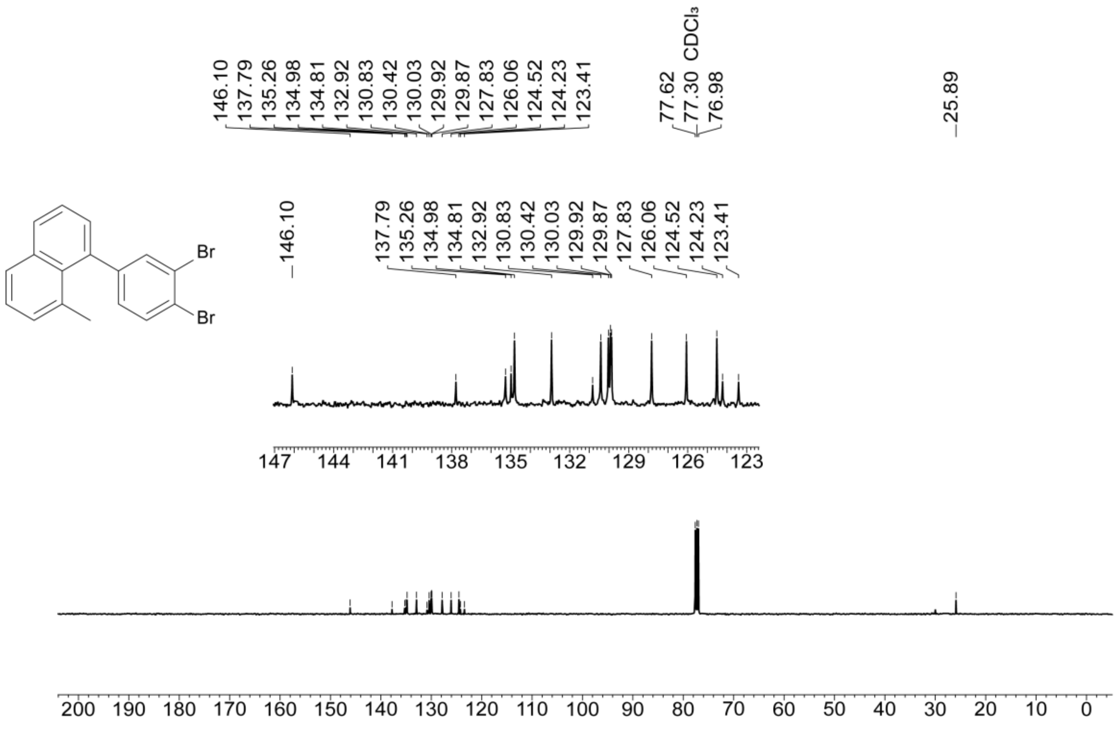

Supplementary Figure 14. Liquid-state ^{1}C NMR spectrum of compound **1** measured in $CDCl_3$ at room temperature. Frequency: 101 MHz.

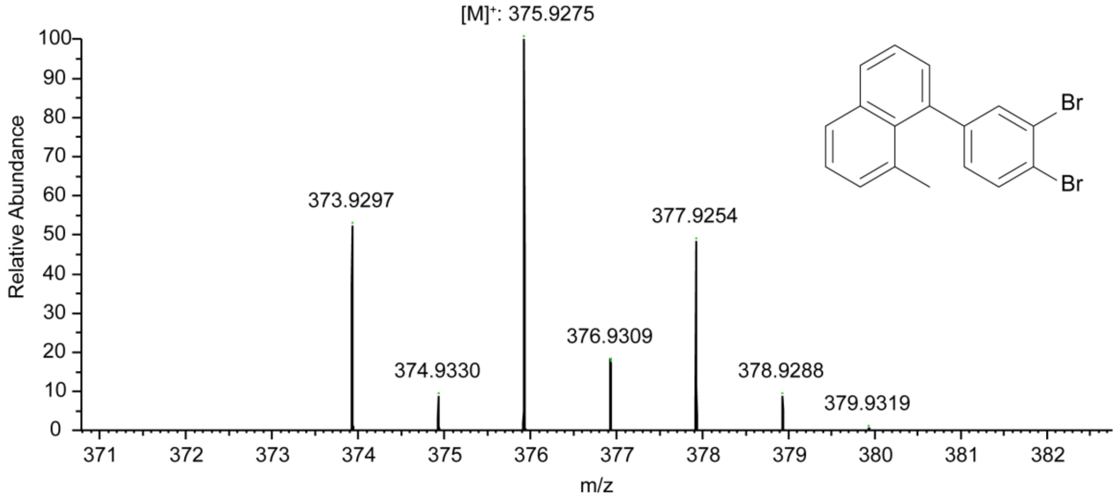

Supplementary Figure 15. Liquid-state HR-APCI-MS (positive mode) of compound **1**.

2 Extended electronic characterization data

2.1 d*I*/d*V* and d^2I/dV^2 spectra of D1 and D2

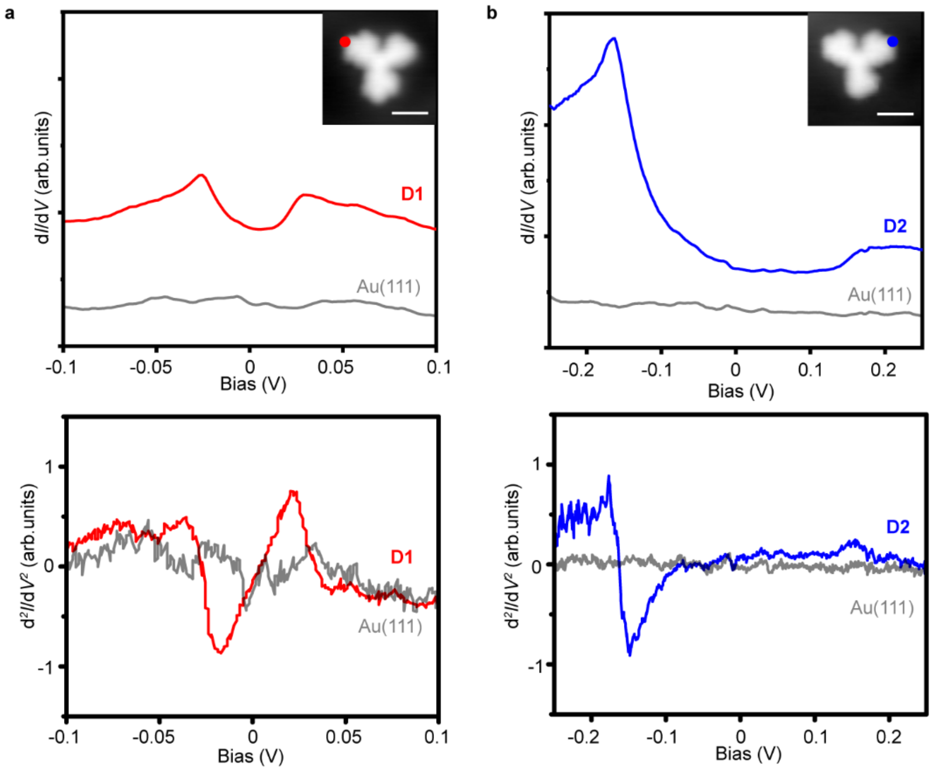

Supplementary Figure 16. The inelastic spin excitation of **D1** and **D2**. **a** The d*I*/d*V* (V = 100 mV, I = 200 pA, V_{rms} = 5 mV) and corresponding d^2I/dV^2 spectra acquired on **D1**, revealing inelastic spin excitation at ±20 meV. **b** The d*I*/d*V* (V = 250 mV, I = 400 pA, V_{rms} = 5 mV) and corresponding d^2I/dV^2 spectra acquired on **D2**, revealing inelastic spin excitation at ±160 meV. Scale bar : 1nm.

2.2 Large range dI/dV spectra and dI/dV mappings of D1 and D2 and T

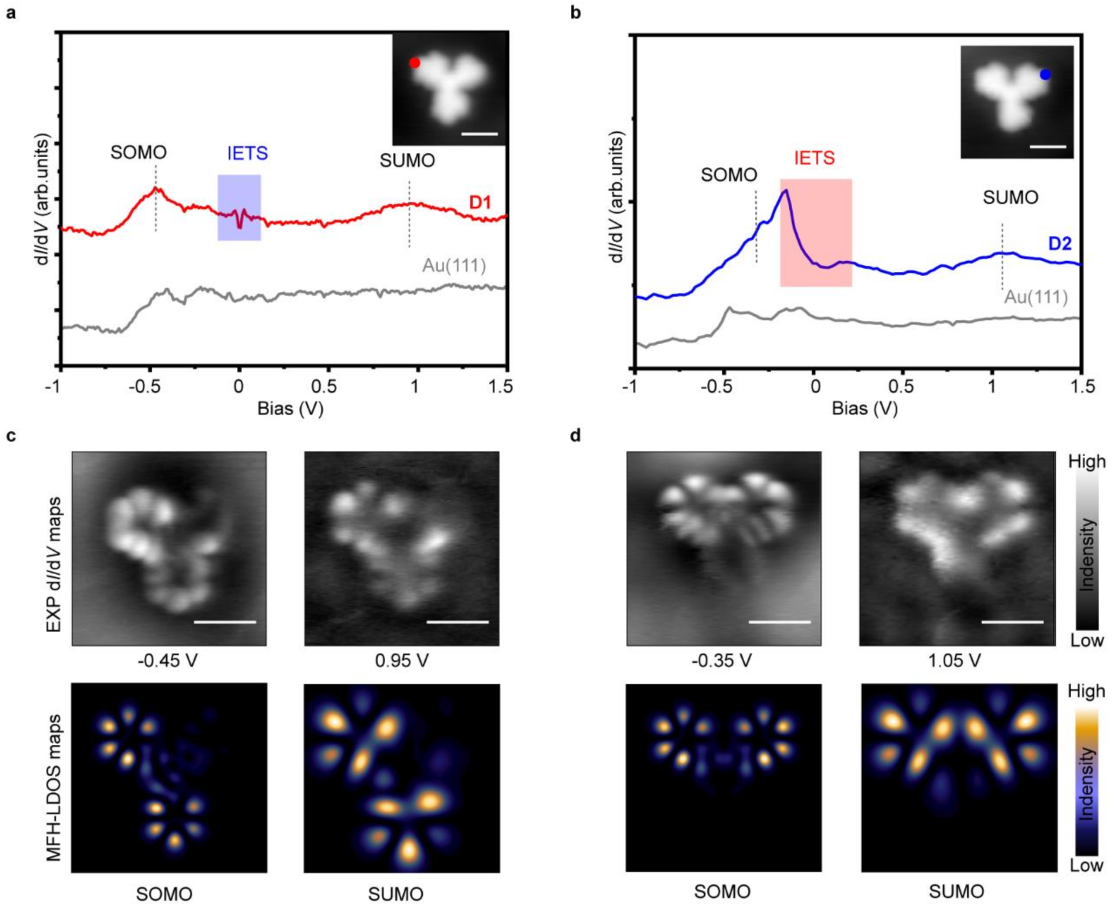

Supplementary Figure 17. Large range dI/dV spectra of **D1** and **D2**. **a** dI/dV (V = 1.5 V, I = 200 pA, V_{rms} = 5 mV) spectra acquired on **D1**. Inset : STM image (V = 300mV, I = 50 pA) of **D1**. **b** dI/dV (V = 1.5 V, I = 200 pA, V_{rms} = 5 mV) spectra acquired on **D2**. Inset : STM image (V = 300 mV, I = 50 pA) of **D2**. **c** dI/dV maps acquired at peak labeled as SOMO (I = 500 pA, V_{rms} = 5 mV) and SUMO (I = 400 pA, V_{rms} = 5 mV) in **a**. The simulated SOMO and SUMO maps are taken at the tip-sample distance of 1.4 Å and 5.7 Å respectively. **d** dI/dV maps acquired at peak labeled as SOMO (I = 500 pA, V_{rms} = 5 mV) and SUMO (I = 400 pA, V_{rms} = 5 mV) in **b**. The simulated SOMO and SUMO maps are taken at the tip-sample distance of 1.4 Å and 5.7 Å respectively. Scale bars : 1 nm

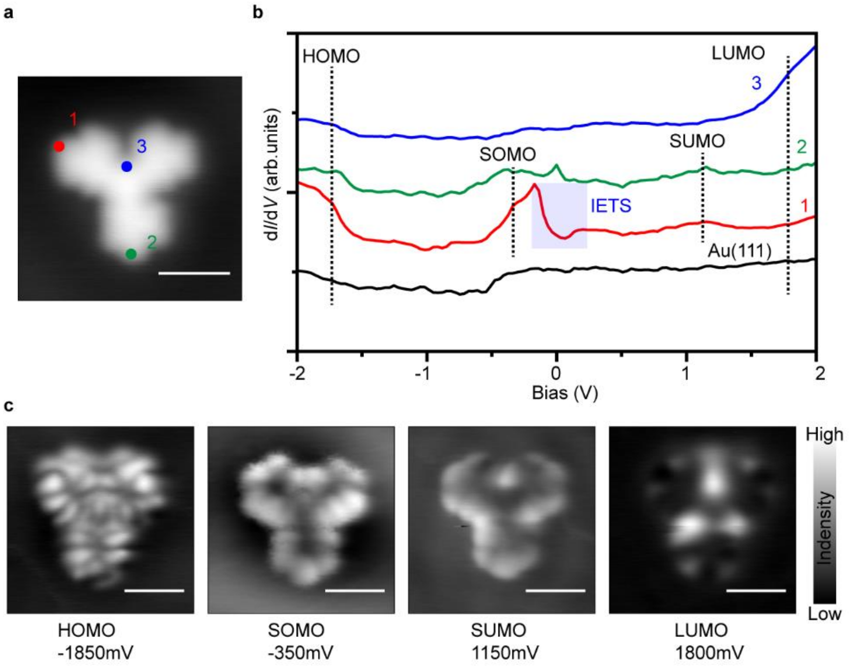

Supplementary Figure 18. Large range dI/dV spectra of **T**. **a** STM image (V = 300 mV, I = 50 pA) of **T**. **b** The dI/dV (V = 1.5 V, I = 200 pA, V_{rms} = 5 mV) spectra on **T**. Acquisition positions are marked with corresponding filled circles in **a**. **c** dI/dV maps acquired at peak labeled as HOMO (I = 500 pA, V_{rms} = 5 mV), SOMO (I = 200 pA, V_{rms} = 5 mV), SUMO (I = 1000 pA, V_{rms} = 5 mV) and LUMO (I = 400 pA, V_{rms} = 5 mV) in **b**. Scale bar: 1nm.

3 Extended theoretical calculations

3.1 Magnetic ground state and first excited spin state of D1 and D2 using MFH method

Supplementary Table 1. Energies of open-shell triplet states with respect to the open-shell singlet ground state (GS) of **D1** and **D2** calculated with MFH method with different t_3.

	D1				D2			
	t_3 = 0 eV		t_3 = -0.4 eV		t_3 = 0 eV		t_3 = -0.4 eV	
	S = 0	S = 1	S = 0	S = 1	S = 0	S = 1	S = 0	S = 1
E (eV)	-235.9938	-235.9777	-229.1311	-229.1047	-236.6709	-236.6082	-229.7906	-229.6922
$E_{triplet}$ - $E_{singlet}$ (meV)	16.1		26.4		62.7		98.4	

3.2 Effect of next-nearest hopping term t_2

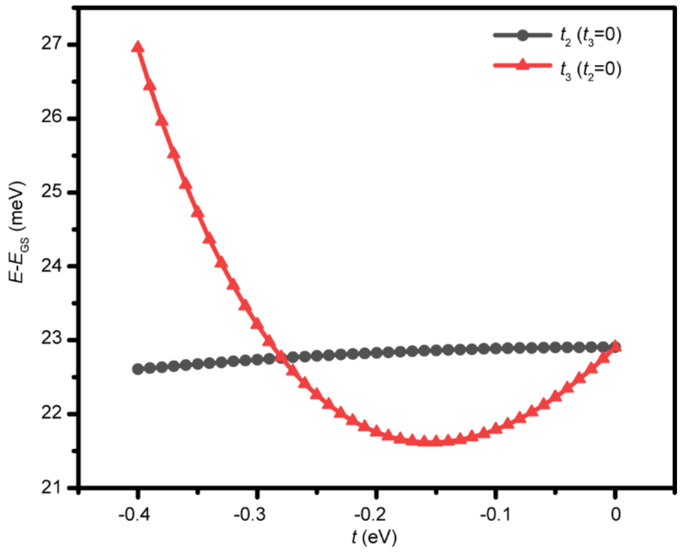

Supplementary Figure 19. Energies of open-shell triplet states with respect to the open-shell singlet ground state (GS) vary with different values of t_2 (in which case $t_3 = 0$ eV) and t_3 (in which case $t_2 = 0$ eV)

3.3 Wave function distribution calculated with U = 0 eV and U = 3.5 eV

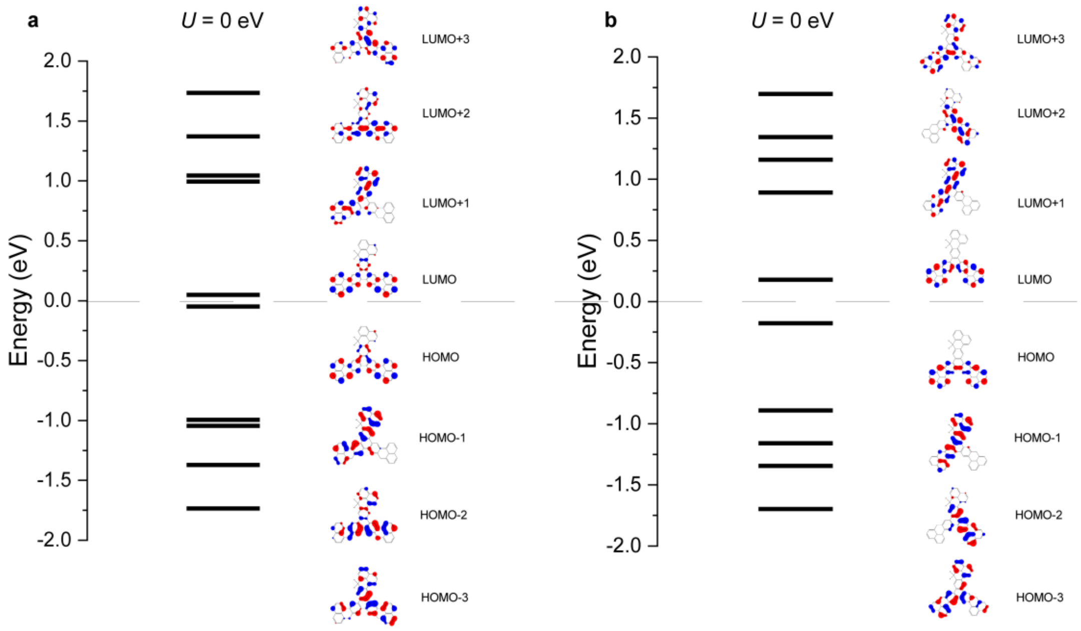

Supplementary Figure 20. a Wave function distribution calculated with U = 0 eV of **D1**. **b** Wave function distribution calculated with U = 0 eV of **D2**.

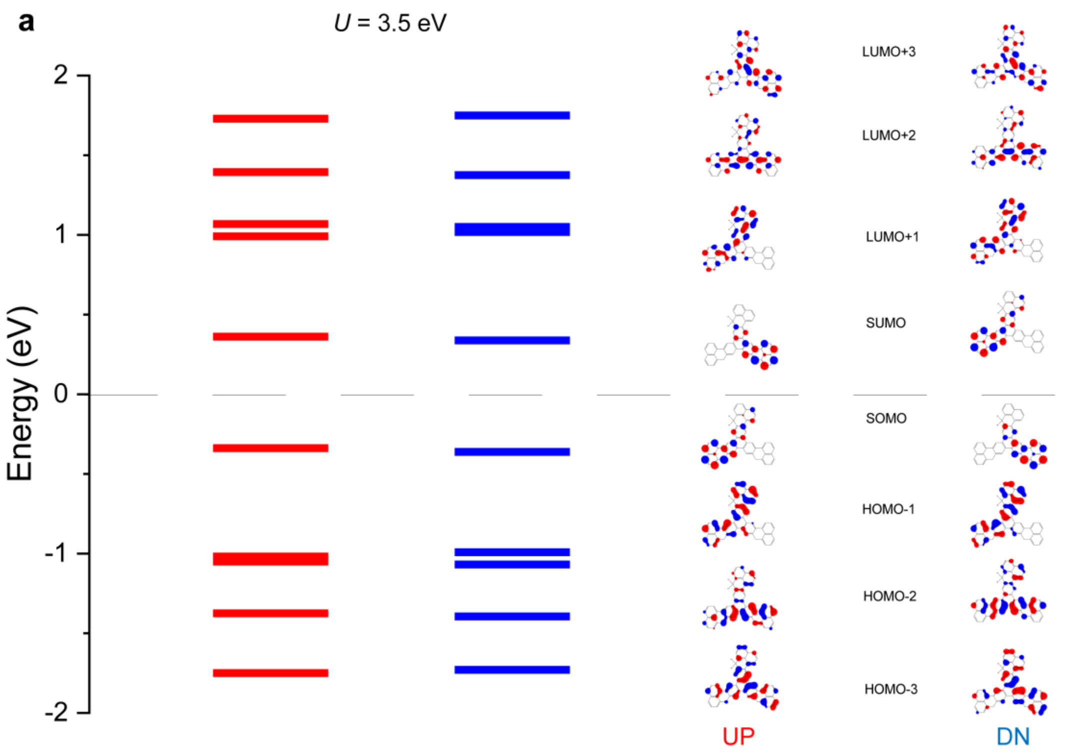

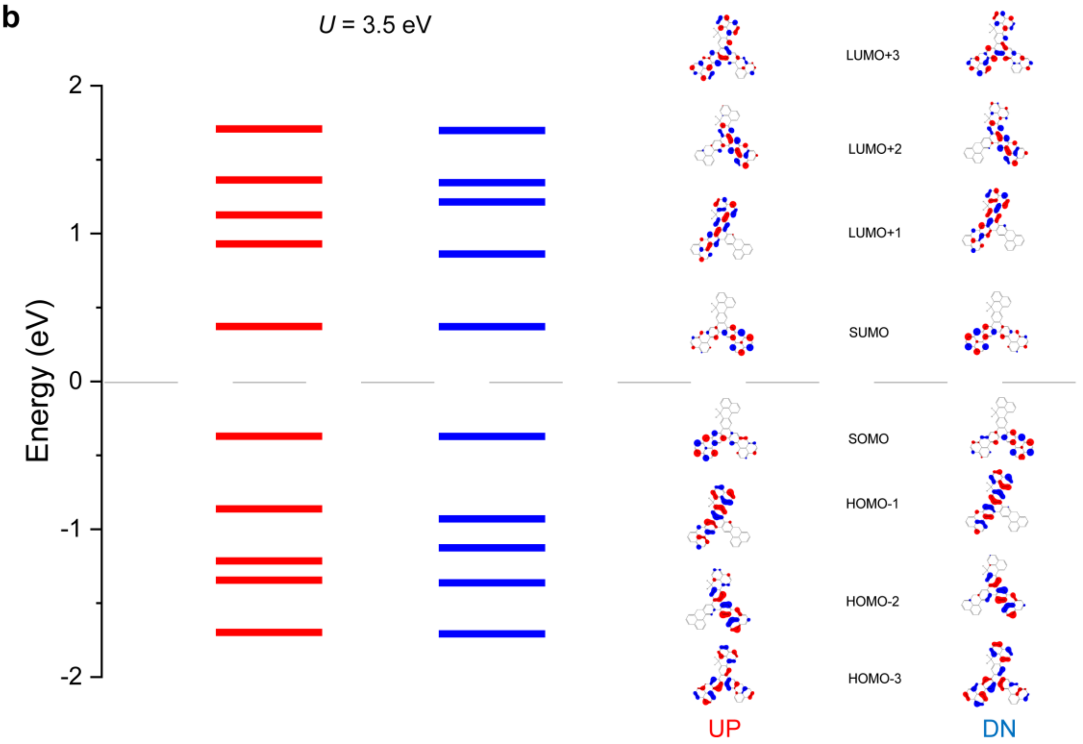

Supplementary Figure 21. a Wave function distribution calculated with *U* = 3.5 eV of **D1**. **b** Wave function distribution calculated with *U* = 3.5 eV of **D2**.

4 Sample preparation and AFM, STM, STS measurements.

The STM/AFM experiments for the electronic and chemical structure characterization were performed at 4.7 K with commercial Createc LT-STM/qplus AFM. The Au(111) single-crystal was cleaned by cycles of argon ion sputtering and subsequently annealed to 800 K to get atomically flat terraces. Molecular precursors **1** were thermally deposited on the clean Au(111) surface, and subsequently annealed to 433 K and 523K to fabricate structure **2** and **3** , The AFM measurements were performed with the qPlus sensor with the resonance frequency of 32.6 KHz and the oscillation amplitude of 50 pm. d*I*/d*V* measurements were performed with an internal lock-in amplifier at frequency of 862 Hz. Lock-in modulation voltages for individual measurements were provided in the respective figure caption. All STM/STS and AFM measurements were acquired with CO-functionalized tungsten tip. For the constant-height AFM images, the tip-distance is decreased a few hundred of pm from the STM set point $V = 300$ mV, $I =$ 50 pA.

5 Supplementary References

1. Su, X. et al. Atomically precise synthesis and characterization of heptauthrene with triplet ground state. *Nano Lett.* **20,** 6859–6864 (2020).
